# Society Meetings

**Published:** 1893-06

**Authors:** 


### Society Meetings.

### AMERICAN SURGICAL ASSOCIATION.

The programme of the annual meeting to be held in the new Alumni Hall, of the Medical Department of the University of Buffalo, 20 High street, Buffalo, N. Y., May 30, 31, June 1, 1893. Members of the medical profession are cordially invited to attend any of the meetings of the association, save when it is in executive session.

May 30th, Tuesday.—Session at 10 a. m.

Roll call.

Address of Welcome, by Prof. M. D. Mann, Dean of the Medical Department, University of Buffalo; President's Address, A New Method of Direct Fixation of Fragments in Compound and Ununited Fractures; Discussion by Drs. Roswell Park, F. S. Dennis, and De Forrest Willard; Pneumotomy for Removal of a Horse-shoe Nail from the Right Lung of a Child, by W. T. Briggs, M. D., Nashville, Tenn. Executive Session.

*Afternoon Session, 2 o'clock:* Hypertrophies and Degenerations of Cicatrices and Cicatricial Tissue, by Dr. J. Collins Warren, Boston; Discussion by Drs. C. H. Mastin, G. R. Fowler, and W. H. Carmalt; Surgery of the Gall Bladder, by Dr. M. H. Richardson, Boston; Discussion by Drs. J. Ewing Mears, A. Vander Veer, W. H. Carmalt, and Theo. A. McGraw.

WEDNESDAY MORNING.—SESSION AT 10 A. M.

At the Buffalo General Hospital, 100 High street. Exhibition of Cases and Specimens; presentation of volunteer papers, should time permit.

*Afternoon Session, 2 P. M.:*—Alumni Hall. Surgical Treatment of Cervical, Thoracic, and Abdominal Aneurism, by Dr. C. B. Nancrede, Ann Arbor; Surgery of the Rectum, by Dr. A. G. Gerster, New York; Discussion by Drs. L. S. Pilcher, H. H. Mudd, and L. McLane Tiffany. Executive Session at 5 P. M.

THURSDAY MORNING.—SESSION AT 10 A. M.

Executive Session. Surgery of the Prostate, by Dr. J. William White, Philadelphia; Discussion by Drs. Hunter McGuire, T. F. Prewitt, R. F. Weir, and F. H. Gerrish; Treatment of Carbuncle, by Dr. F. Lange, New York; Discussion by Drs. Robert Abbe, J. B. Roberts, and J. S. Wight.

In addition to the above, the following papers are volunteered and will be read, as opportunity offers, at the end of each day's programme:

Unreduced Dislocations of the Astragalus, by Dr. Stephen Smith, New York; Clinical Reports: Two Cases of Primary Sarcom of the Tonsil, with Operation; Cases of Operation upon Meckel's and the Gasserian Ganglions; Malignant Polyp, springing from the base of the skull; attempt to make the operation bloodless, after Senn's suggestion, by Dr. Roswell Park, Buffalo; The Importance of the Colon Bacillus to the General Surgeon, by Dr. Roswell Park.

President, Dr. Nicholas Senn, Chicago; Vice-Presidents, Dr. W. W. Keen, Philadelphia, Dr. Charles B. Porter, Boston; Secretary, Dr. J. R. Weist, Richmond, Ind.; Treasurer, Dr. J. B. Roberts, Philàdelphia; Recorder, Dr. J. Ewing Mears, Philadelphia; Council, Dr. Robert F. Weir, New York; Dr. F. S. Dennis, New York; Dr. Stephen H. Weeks, Portland, Me.; Dr. C. H. Mastin, Mobile; Dr. Roswell Park, Buffalo; Chairman of Committee of Arrangements, Dr. Roswell Park.

---

### AMERICAN MEDICAL ASSOCIATION.

PROGRAMME of the Section of Medicine, Milwaukee, Wis., June 6 to 9, 1893.

MORNING OF JUNE 6TH.

Address of the Chairman. A Review of Incurative Endocarditis, Dr. Chas. G. Stockton, Buffalo, N. Y.; The Complications of Chronic

Nephritis and Their Treatment, Dr. Wm. C. Dabney, University of Virginia; To Bleed or not to Bleed in Pneumonia, Dr. John North, Toledo, O.; Some Considerations Bearing on the Treatment of Pneumonia, Dr. W. H. Washburne, Milwaukee, Wis.; The Medical Aspects of Empyema, Dr. Robt. H. Babcock, Chicago, Ill.; Some Thoughts on the Differential Diagnosis of Chest Disease, Dr. J. G. Truax, New York City; Discussion, Dr. J. A. Larrabee, Louisville, Ky.

AFTERNOON OF JUNE 6TH.

A Case of Sunstroke with Considerations of its Treatment, Dr. Joseph Eichberg, Cincinnati, O.; The Treatment of Insolation, Dr. R. R. Ross, Buffalo, N. Y.; The Treatment of Pyrexia, Dr. James Witherspoon, Columbia, Tenn.; Demonstration of Gastrodiaphany, Dr. Max Einhorn, New York City; The New Chemistry of the Stomach as a Means of Diagnosis and a Guide to Therapeutics, based upon the Results of One Thousand Analyses of Stomach Fluids, Dr. J. H. Kellogg, Battle Creek, Mich.; The Value of Belladonna and Strychnine in Conditions of Circulatory Depression, Dr. H. A. Hare, Philadelphia, Pa.; Points on the Clinical History of Erysipelas, Dr. J. M. Andus, Philadelphia, Pa.

MORNING OF JUNE 7TH.

The Management of Hysteria by the General Practitioner, Dr. W. F. Rochelle, Jackson, Tenn.; Headache, Dr. James W. Putnam, Buffalo, N. Y.; An Additional Note on the Treatment of Exophthalmic Goitre, Dr. E. D. Ferguson, Troy, N. Y.; Hematoma Auris, Dr. H. A. Matzinger, Buffalo, N. Y.; Aneurisms Considered Medically Rather than Surgically, Dr. O. C. McNary, Leavenworth, Kan.; Insanity of Physiological Epochs, Dr. A. W. Hurd, Buffalo, N. Y.

AFTERNOON OF JUNE 7TH.

The Treatment of Cholera, Dr. J. H. Hollister, Chicago, Ill.; Brief Clinical Memoranda. (*a*) An Available Catheter Lubricant, (*b*) Facile Reduction of Prolapsed Hemorrhoids, (*c*) Preparing Hemorrhoids for Injection, (*d*) Ready Relief for Lumbago, Dr. H. A. Didama, Syracuse, N. Y.; Medical Treatment of Typhlitis and Appendicitis, Dr. F. W. Ross, Elmira, N. Y.; An Unusual Case of Appendicitis, Dr. J. H. Musser, Philadelphia, Pa.; Discussion of Dr. Musser's Case and Appendicitis, Dr. Joseph Price, Philadelphia, Pa.

MORNING OF JUNE 8TH.

A Case of Cancer en Cuirasse, with Report of an Autopsy and Illustrations, Dr. J. B. Marvin, Louisville, Ky.; The Cancer Parasite from a Micro-technical Standpoint, Dr. A. P. Ohlmacher, Chicago, Ill; The Association of Diseases and Morbid Processes, Dr. H. A. West, Galveston, Texas; Studies in Immunity, Dr. D. N. Kinsman, Columbus, O.; Early Aspiration in Acute Pleuritis, Dr. E. J. C. Minard, Brooklyn,

N. Y.; A Preliminary Report on an Inquiry as to the Frequency of a Form of Kidney Degeneration in which Albuminuria may not be Regarded as a Symptom.

AFTERNOON OF JUNE 8TH.

The Origin and Clinical Significance of Uric Acid, Dr. Victor C. Vaughan, Ann Arbor, Mich.; The Brand Method of Treating Typhoid Fever, Dr. James C. Wilson, Philadelphia, Pa.—This Topic will be discussed by: (*a*) Dr. Chr. Sihler, Cleveland, O., (*b*) Dr. H. A. LaFleur, Montreal, Que., (*c*) Dr. Geo. Wilkins, Montreal, Que., (*d*) Dr. L. Wolff, Philadelphia, Pa., (*e*) Dr. W. Gilman Thompson, New York, N. Y., (*f*) Dr. Wm. Pepper, Philadelphia, Pa.; Cold Water in Typhoid Fever, Dr. H. H. Middlekamp, Warrenton, Mo.; The Treatment of Intestinal Hemorrhage in Typhoid Fever, with Cases, Dr. Z. J. Lusk, Warsaw, N. Y.

MORNING OF JUNE 9TH.

Experiences with Trional as a Hypnotic, Dr. W. C. Krauss, Buffalo, N. Y.; One Thousand Cases of Typoid Fever, Dr. James B. Herrick, Chicago, Ill.; The Present Stand of Tuberculin, Dr. J. T. Whittaker, Cincinnati, Ohio; The Mutual Interest of the Medical Profession and Insurance Companies in the Prolongation of Human Life, Dr. Chas. Denison, Denver, Colo.; Typhoid Fever, Dr. M. J. Crouch, Union, Ky.

AFTERNOON OF JUNE 9TH.

A Case of Fever of Undetermined Type, Dr. Starling Loving, Columbus, Ohio; Some Remarks on Diabetes, Dr. N. S. Davis, Chicago, Ill.; The Detection and Significance of Carbohydrates in the Urine, Dr. Chas. W. Purdy, Chicago, Ill.; The Treatment of Diabetes Mellitus by Jambul (Syzygium Jambolaneum), Dr. James Tyson, Philadelphia, Pa.; Heat in the Treatment of Disease, Dr. Chas. H. Shephard, Brooklyn, N. Y.

---

AMERICAN ACADEMY OF MEDICINE.—Preliminary programme of the meeting for 1893, at Milwaukee.

*Saturday Morning, June 3d, at 10 o'clock.*—Executive Business; Reports of Committees; On Eligible Fellows; On the Requirements for Preliminary Education in the Various Medical Colleges in the United States and Canada; On the Comparative Value of Academical Degrees. Papers: The Attitude of our Medical Schools in Relation to Preliminary Studies, R. Lowry Sibbet, Carlisle, Pa. What Mental Faculties Should be Specially Trained for the Study of Medicine? James W. Moore, Lafayette College. The Classics and the Common Schools, J. Berrien Lindsley, Nashville, Tenn.

*Saturday Afternoon at 2.30.*—President's Address. Papers: What Should be Required in an Entrance Examination to a Medical School?

James W. Holland, Jefferson Medical School. Should there be Elective Studies in a Medical Course? P. S. Conner, Medical Department, Dartmouth College. On the Endowment of Medical Schools, George M. Gould, Philadelphia.

Re-union Session, Saturday evening.

*Monday Morning, June 5th, at 10 o'clock.*—Executive Business; Report of Committee on the Laws Regulating the Practice of Medicine. Papers: The Duty of the State to Medicine, Benjamin Lee, Philadelphia. The Importance of the Study of Medical Sociology, Charles McIntire, Easton, Pa. Title to be announced later—C. C. Bombaugh, Baltimore.

There will probably be time at the meeting for the reading of two or three papers more than are now on the programme. Fellows who desire to contribute such papers are requested to send the titles to the Secretary. Some of the papers are promised provisionally and are announced with this understanding.

---

The American Medical Association meets at Milwaukee, Wis., June 6, 7, 8, 9, 1893.

---

The American Microscopical Society and the American Association for the Advancement of Science meet at Madison, Wisconsin, August 14, 15, and 16, 1893.

---

The next meeting of the Buffalo Microscopical Club will be held in the Library Building, Monday evening, June 19, 1893. This being the annual meeting, the president's address will be given; subject, Nuclear Origin of the Cranial Nerves, by William C. Krauss, M. D. Report of officers and election of officers will follow.

---

The semi-annual meeting of the Erie County Medical Society will be held Tuesday, June 13, 1893, at 10 A. M., in the rooms of the Buffalo Academy of Medicine, Market Arcade. The committee has arranged the following excellent programme: The Use of the Metric System. Discussion opened by Dr. A. L. Benedict, chairman of the committee on the metric system. Discussion on Renal Insufficiency; diagnosis by Dr. A. A. Jones; treatment by Dr. DeLancey Rochester.

---

The twenty-sixth annual meeting of the Medical Association of Central New York will be held in the Chamber of Commerce,

